# Tonga national emergency medical team response to the 2022 Hunga Tonga-Hunga Ha’apai volcanic eruption and tsunami: the first deployment of the Tonga Emergency Medical Assistance Team (TEMAT)

**DOI:** 10.5365/wpsar.2023.14.6.1026

**Published:** 2023-05-18

**Authors:** Siosifa Sifa, Sela Ki Folau Fusi, Sean T Casey, Penisimani Poloniati, Kaloafu Tavo, Yutaro Setoya, Ana, Akauola

**Affiliations:** a World Health Organization Country Liaison Office for Tonga, Nuku’alofa, Tonga.; b Global Center for Preventive Health and Nutrition (GLOBE), Faculty of Health, Deakin University, Burwood, Victoria, Australia.; c World Health Organization Regional Office for the Western Pacific, Manila, Philippines.; d School of Population Health, University of New South Wales, Sydney, New South Wales, Australia.; e Tonga Ministry of Health, Nuku’alofa, Tonga.

## Abstract

**Problem:**

The undersea Hunga Tonga-Hunga Ha’apai volcano erupted on 15 January 2022, causing a tsunami that affected Tonga as well as other countries around the Pacific rim. Tonga’s international borders were closed at the time due to the coronavirus disease pandemic, but clinical surge support was needed to respond to this disaster.

**Context:**

Tonga’s Ministry of Health formed the Tonga Emergency Medical Assistance Team (TEMAT) in 2018 to provide clinical care and public health assistance during disasters, outbreaks and other health emergencies. TEMAT was activated for the first time in January 2022 to respond to medical and public health needs following the eruption and tsunami.

**Action:**

On 16 January 2022, a five-person TEMAT advance team was deployed to conduct initial damage assessments and provide casualty care. Subsequently, TEMAT rotations were deployed to provide clinical care and public health support across the Ha’apai island group for over 2 months.

**Outcome:**

TEMAT deployed to the islands most affected by the volcanic eruption and tsunami within 24 hours of the event, providing emergency clinical, psychosocial and public health services across four islands. TEMAT reported daily to the Ministry of Health and National Emergency Management Office, providing critical information for response decision-making. All TEMAT actions were documented, and an after-action review was conducted following the deployment.

**Discussion:**

TEMAT’s deployment in response to the 2022 volcanic eruption and tsunami highlighted the importance of national emergency medical teams that are prepared to respond to a range of emergency events.

On 15 January 2022, the undersea Hunga Tonga-Hunga Ha’apai (HTHH) volcano erupted violently, triggering an unprecedented plume of smoke and ash, and a tsunami that struck low-lying coastal areas of Tonga’s most populous island of Tongatapu, the nearby island of ’Eua, and multiple islands in the country’s Ha’apai island group, in addition to other countries around the Pacific rim. (*1*, *2*) Thick volcanic ash covered nearly the entire kingdom, and the health impacts were initially expected to be significant. The country’s undersea internet cable was damaged, cutting off nearly all domestic and international communications. Tonga’s Prime Minister declared a State of Emergency, although, with communication channels inoperable, the needs in the kingdom’s outer islands were not initially known. The Tonga Emergency Medical Assistance Team (TEMAT) was activated by Tonga’s Ministry of Health to support the health response in the Ha’apai island group. This report describes TEMAT’s activities and documents the lessons learned during the response.

## CONTEXT

Tonga is an island nation of approximately 100 000 people located in the South Pacific with 172 islands, of which 45 are inhabited, spread across four archipelagos covering 700 000 km^2^ of ocean territory. (*3*) As an island kingdom with a high proportion of its population in low-lying coastal areas, Tonga is particularly vulnerable to natural hazards and climate change impacts, including tropical cyclones, earthquakes, tsunamis, flooding and volcanic eruptions. (*4*) In 2018, the World Risk Index ranked Tonga as the second most at-risk country in the world, after Vanuatu. (*5*)

Recognizing the persistent threat of disasters and outbreaks, particularly with the potential to affect the country’s outer islands, Tonga’s Ministry of Health established TEMAT as the kingdom’s national emergency medical team (EMT) in 2018. (*6*) This was accomplished with technical and operational support from the World Health Organization (WHO), and with funding and technical support from the governments of Australia and New Zealand. TEMAT is located in Tonga’s Ministry of Health, with coordination linkages to Tonga’s National Emergency Management Office and National Emergency Management Committee. TEMAT was established based on the principles and standards detailed in the WHO *Classification and minimum standard for foreign medical teams in sudden onset disasters* (updated in 2021 to *Classification and minimum standards for emergency medical teams* (*7*)), with adaptations to account for Tonga’s small size, limited human resources and unique island context.

The aim of TEMAT is to provide clinical care and public health assistance to populations affected by disasters, outbreaks and other health emergencies. TEMAT consists of physicians, nurses, pharmacists, environmental health officers and operations support and logistics personnel. TEMAT developed standard operating procedures (SOPs) and received a cache of equipment and supplies from WHO to ensure self-sufficiency in deployment (**Table 1**). TEMAT members had been trained by WHO and regional experts in 2018 and 2019 but had not been deployed in response to a disaster until the HTHH volcanic eruption and tsunami in January 2022.

**Table 1:** TEMAT deployment cache

Item	Quantity
Personal deployment kit (including camping supplies such as headlamps, sleeping bags, mats, torches, clear safety glasses, tarpaulins, etc.)	16
First aid kit	16
Medical backpack with supplies provided by Tonga Central Pharmacy	8
Accommodation tent (four-person tent)	4
Community LifeStraw® water filtering system	4
N95® mask	5 boxes
Generator EU10i (1kVA)	1
Drum of diesel (200 litres)	3
Food and cooking supplies (including gas stoves, gas bottles, pots, plates, bowls and utensils)	Food supplies for 1 week with regular replenishment from Tongatapu
Water (bottled water for team members)	50 packs
Mist blower for vector control	3
Chemical for insecticide spraying (Aqua K-Othrine® and bifenthrin)	6 bottles (4 litres each)
Satellite phone (provided by the World Bank office, Tonga)	1

## ACTION

**Fig. 1:**
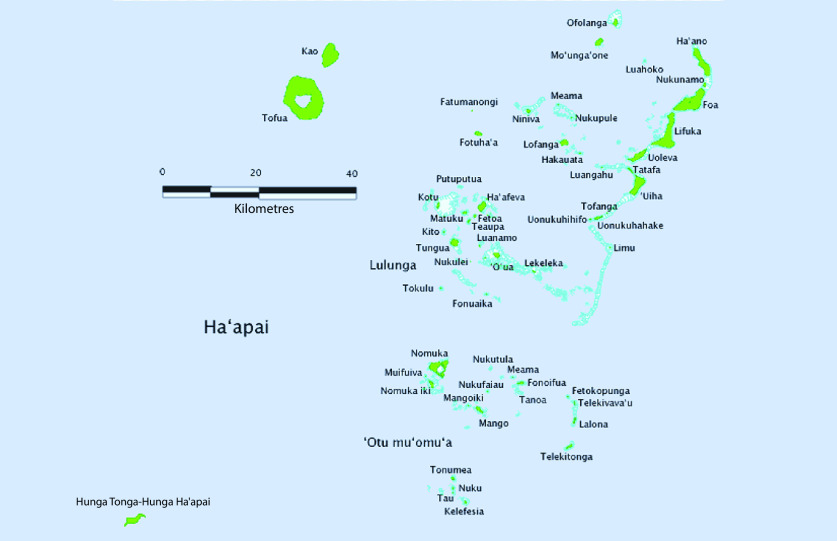
Map of Ha’apai islands indicating the islands to which TEMAT responded

Tonga’s National Emergency Management Committee convened an emergency meeting on the night of the HTHH eruption on 15 January 2022. (*1*) A multidisciplinary team was deployed on 16 January to the Ha’apai islands (**Fig. 1**) aboard His Majesty’s Armed Forces (HMAF) Guardian-class patrol ship, the VOEA Ngahau Koula. This deployment included a five-person TEMAT advance team consisting of two physicians and three nurses, (*8*) tasked with undertaking an initial damage/needs assessment and caring for casualties. As communication with the Ha’apai islands had not been re-established, the level of damage and needs were unknown. (*2*)

[**insert** Figure 1]

The Ngahau Koula reached the island of Nomuka on the morning of 17 January, after sailing for approximately 20 hours, with the ship encountering delays due to significant debris in the sea following the tsunami. Enormous destruction from the tsunami was observed, with nearly all coastal dwellings destroyed, including Nomuka’s local health centre. TEMAT assessed that there was a comparably small number of injuries, which they were able to treat on-site in a makeshift clinic, with the medicines and clinical supplies they had brought with them. One patient was evacuated to the national referral hospital in Tongatapu, and one death from the tsunami was recorded from Nomuka.

The five-person TEMAT team was subsequently divided into two teams, with one supporting clinical service delivery on Nomuka and the other travelling on board the HMAF ship to other Ha’apai islands to continue assessments. The people of Ha’apai’s Mango island sighted the HMAF ship and communicated with it using a signal mirror. Upon TEMAT’s arrival onshore, the island’s population was sitting in the open on a hillside as the tsunami had destroyed every structure on the island. On 21 January, another team was deployed with four members: one medical officer and three emergency nurses. They assisted Tonga’s Armed Forces to evacuate the entire population of Mango (*n* = 62) to Tongatapu and ‘Eua.

While the first TEMAT rotation was in Ha’apai, a second, larger team was mobilized and deployed to Nomuka to ensure continuity of services for the affected population there, including clinical care and public health action. The composition of this team was decided based on feedback received from the first TEMAT rotation, reported via satellite phone to health authorities in Tongatapu. The second TEMAT rotation departed Tongatapu late on 18 January with eight team members: two medical officers, two clinical nurses, one health inspector, one pharmacist and two logisticians (water and sanitation officers). Additional TEMAT rotations followed during February and early March 2022 and were based on TEMAT’s initial needs assessment. TEMAT was demobilized in March 2022, following a 7-week deployment to Ha’apai.

An after-action review (AAR) of the deployment was conducted in December 2022, which was delayed due to the coronavirus disease (COVID-19) response that was initiated soon after the volcanic eruption and tsunami. (*9*) The AAR was conducted using a hybrid workshop, which allowed for both virtual and in-person attendance. The attendees included TEMAT’s chairperson, medical officers and nurses who were part of the deployments, as well as WHO regional EMT experts. The AAR provided valuable insights into the response effort, documented lessons learned and highlighted areas for improvement to ensure better preparedness and response in the future.

## OUTCOME

TEMAT’s response was launched within 24 hours of the volcanic eruption and tsunami, reaching the worst-affected islands within approximately 40 hours. TEMAT provided emergency clinical service delivery, psychosocial support and public health response to the most affected communities of the Ha’apai islands, caring for 381 patients across four islands (**Fig. 1; ****Table 2**). TEMAT’s actions included emergency care for a small number of trauma patients, but also extensive action to manage patients with chronic noncommunicable diseases, replace lost medications, provide medical evacuation assistance and support local nurses to re-establish clinical services on the islands. They also conducted a range of environmental health actions, including water treatment, vector-control spraying and debris removal.

**Table 2:** Health-care services TEMAT provided to patients in Ha’apai (Fonoifua, Ha’afeva, Nomuka, Mango)

Health-care services	No. of patients
Treatment for noncommunicable diseases (monitoring vital signs, dispensing medication, lifestyle advice)	297
Psychosocial support (advice and counselling)	39
Treatment for communicable diseases (monitoring vital signs, sample collection, diagnostic testing, dispensing medication, advice on preventive measures)	27
Treatment for traumatic injuries (wound care and dressing, intravenous medication and fluid resuscitation)	13
Transfers to the national referral hospital	5

TEMAT reported to the Ministry of Health and National Emergency Management Office through a satellite phone, with reports conducted on a daily basis to provide critical information for decision-making at the national level.

The key lesson from TEMAT’s deployment in response to the 2022 HTHH volcanic eruption and tsunami is the importance of having national EMT capability, regardless of a country’s size. While Tonga is one of the world’s smallest countries by population, having a trained, equipped and self-sufficient EMT that is rapidly deployable to remote locations is essential for health emergency response, particularly for countries with high vulnerability to natural and infectious hazards and challenging geography.

EMTs must be ready to respond at short notice, with the required equipment and documented procedures on hand. The availability of TEMAT SOPs and EMT trainings in 2018 and 2019 contributed to the team’s deployment readiness. Having the EMT cache on-hand at the time of TEMAT’s deployment ensured that team members were able to function safely, effectively and self-sufficiently in Ha’apai, even after the complete destruction of local health infrastructure. The fact that TEMAT was ready for rapid deployment with the appropriate cache was a key success factor, underscoring the importance of making these investments before disasters occur.

However, despite having a prepared EMT cache, the first TEMAT rotation departed without the full kit of equipment and supplies, as there was very little time to make arrangements before departure, and because communication lines were down at the time. Some team members departed with quickly packed bags of their own clothes and limited food, though HMAF provided rations for everyone deployed on the first mission to Ha’apai on 16 January. A satellite phone provided by the World Bank office in Tonga facilitated communication between TEMAT and the Ministry of Health in Tongatapu. However, as this was the only method of communication, reporting to the Ministry of Health was limited to daily calls via the satellite phone. It was not possible to report standard surveillance data collection, and the WHO EMT Minimum Data Set was not used in this response.

Given the severity and extensive impact of the volcanic eruption and tsunami, extensive casualties were expected. However, the majority of the TEMAT response was not trauma-related. Instead, their work comprised providing psychosocial support, managing noncommunicable diseases, treating diarrhoeal and skin diseases relating to damaged water and sanitation infrastructure, and repairing damaged infrastructure. Although TEMAT was well prepared to deploy to a post-disaster context, with the expectation of encountering more trauma presentations, the cache taken by the first teams required adjustment in subsequent re-supply shipments. TEMAT’s flexibility, in terms of changing the team structure and roles, was another lesson learned. Just-in-time training on psychological first aid (PFA) provided by WHO’s Country Liaison Officer in Tonga, who is a psychologist by training, helped some team members to care for the many acute stress presentations that they encountered. As all team members were affected, with their homes covered in ash from the volcanic eruption, and some unable to contact family members, they also required support.

While training had been conducted for TEMAT, with 40 members trained in 2018 and 2019, only a small number of those deployed for the HTHH response had been formally trained as TEMAT members before this activation – one of whom was the team leader for most of the deployment period. Significant turnover in the Ministry of Health, as well as competing demands related to COVID-19 preparedness and response, also reduced the pool of trained personnel available to deploy with TEMAT. Some TEMAT members were not aware of its SOPs, reporting procedures and operation of the cache. The need for regular TEMAT member training was another lesson learned from this response.

## Discussion

TEMAT’s deployment to Ha’apai in response to the HTHH eruption and tsunami was an effective response that met the health needs of the affected population. It also presented an opportunity for learning and continuous improvement of TEMAT’s capability. The AAR of TEMAT’s HTHH response identified several lessons and areas for improvement. TEMAT’s SOPs are now under review, including inventory lists for clinical and non-clinical cache, deployment checklists and reporting forms and protocols. A TEMAT training and simulation exercise was carried out in April 2023 with support from WHO, using a tailored Pacific EMT member training package. (*10*) Additional training on PFA and mental health and psychosocial support in emergencies is also planned. TEMAT will receive an additional cache in 2023, sourced and procured by WHO specifically for Pacific EMTs. (*11*) This will include personal deployment supplies for team members, as well as additional medical and communication equipment such as a portable ultrasound machine and satellite communication devices.

TEMAT’s deployment to the Ha’apai islands was a challenging test for one of the first national EMTs to be developed in the Pacific, and it provides evidence that national teams like TEMAT are capable of independent, self-sufficient response to emergencies within their borders. (*12*) TEMAT’s deployment, like all EMT deployments, provides opportunities for learning and continuous improvement, and Tonga’s Ministry of Health and TEMAT members are committed to building on the lessons from the challenging yet rewarding deployment to Ha’apai in response to this large and complex disaster in January 2022.
